# Rapid Multilateral and Integrated Public Health Response to a Cross-City Outbreak of *Salmonella* Enteritidis Infections Combining Analytical, Molecular, and Genomic Epidemiological Analysis

**DOI:** 10.3389/fmicb.2022.772489

**Published:** 2022-05-04

**Authors:** Min Jiang, Chao Yang, Patrick S. L. Kwan, Liping Zhang, Hang Fan, Yujuan Jin, Lifang Sun, Hongyu Chen, Baisheng Li, Qiuxia Chen, Yarong Wu, Yan Guo, Yuanguo Shi, Min Liao, Xiaolu Shi, Jianping Liu, Lijuan Jiang, Rui Cai, Yinhua Deng, Qun Sun, Ruifu Yang, Qiaoli Zhang, Yujun Cui, Qinghua Hu

**Affiliations:** ^1^Shenzhen Major Infectious Disease Control Key Laboratory, Shenzhen Center for Disease Control and Prevention, Shenzhen, China; ^2^Microbiology Laboratory, Dongguan Center for Disease Control and Prevention, Guangdong, China; ^3^State Key Laboratory of Pathogen and Biosecurity, Beijing Institute of Microbiology and Epidemiology, Beijing, China; ^4^Microbiology Laboratory, Longgang District Center for Disease Control and Prevention, Shenzhen, China; ^5^Department of Laboratory Medicine, Shenzhen Children’s Hospital, Shenzhen, China; ^6^Microbiology Laboratory, Guangdong Center for Disease Control and Prevention, Guangdong, China; ^7^Division of Biohazard Inspection and Testing, Shenzhen Institute of Quality & Safety Inspection and Research, Shenzhen, China; ^8^Key Laboratory of Bio-Resource and Eco-Environment of Ministry of Education, College of Life Sciences, Sichuan University, Chengdu, China

**Keywords:** *Salmonella*, serotype Enteritidis, outbreak response, genomic epidemiology, whole-genome sequencing, metagenomics, single-nucleotide polymorphism

## Abstract

On September 21, 2019, the Shenzhen and Dongguan Centers for Disease Control and Prevention received notification of a large cluster of suspected gastroenteritis involving primarily children who sought medical care at hospitals throughout two adjacent cities in China, Shenzhen, and Dongguan. A joint outbreak response was promptly initiated across jurisdictions in a concerted effort between clinical microbiologists, epidemiologists, and public health scientists. Concurrently, multiplex PCRs were used for rapid laboratory diagnosis of suspected cases; epidemiological investigations were conducted to identify the outbreak source, complemented by near real-time multicenter whole-genome analyses completed within 34 h. Epidemiological evidence indicated that all patients had consumed egg sandwiches served on September 20 as snacks to children and staff at a nursery in Dongguan, located near Shenzhen. *Salmonella* Enteritidis was isolated from case-patients, food handlers, kitchenware, and sandwiches with kitchen-made mayonnaise. Whole-genome single-nucleotide polymorphism (SNP)-based phylogenetic analysis demonstrated a well-supported cluster with pairwise distances of ≤1 SNP between genomes for outbreak-associated isolates, providing the definitive link between all samples. In comparison with historical isolates from the same geographical region, the minimum pairwise distance was >14 SNPs, suggesting a non-local outbreak source. Genomic source tracing revealed the possible transmission dynamics of a *S*. Enteritidis clone throughout a multi-provincial egg distribution network. The efficiency and scale with which multidisciplinary and integrated approaches were coordinated in this foodborne disease outbreak response was unprecedented in China, leading to the timely intervention of a large cross-jurisdiction *Salmonella* outbreak.

## Introduction

Salmonellosis is among the most common foodborne infections in China (Liu et al., 2018), with *Salmonella enterica* serotype Enteritidis (*S*. Enteritidis) being identified as the most prevalent serotype among laboratory confirmed nontyphoidal *Salmonella* infections (Ran et al., 2011). Globally, *S*. Enteritidis from shell eggs has been well recognized as the causative agent of large-scale foodborne outbreaks (Lu et al., 2004; Janmohamed et al., 2011; Hormansdorfer et al., 2017). In the United States, despite long identified as a major vehicle for *S*. Enteritidis infections (Braden, 2006), outbreaks associated with shell eggs have continued to occur, including a nationwide outbreak that triggered a recall of 500 million eggs (Kuehn, 2010). Food products of egg origin used as an ingredient during preparation, such as mousse cake (Zhou et al., 2015) and egg sandwiches (Wei et al., 2014; Guo et al., 2015), were frequently implicated as sources of *S*. Enteritidis infections.

In recent years, the depth and breadth of foodborne outbreak investigations have been greatly enhanced by the use of whole-genome sequencing (WGS) analyses, by offering the ultimate subtyping resolution delivered by interrogating entire bacterial genomes (Deng et al., 2016). As a result, WGS has increasingly complemented traditional epidemiological studies during foodborne outbreak investigations to provide definitive genomic evidence linking suspected food sources to infections (Gymoese et al., 2017; Ford et al., 2018; Ung et al., 2019), with real-time applications also being developed (Jackson et al., 2016; Ribot et al., 2019). In several multijurisdictional and international *Salmonella* outbreaks, WGS has been successfully utilized for elucidating transmission dynamics and traceback investigations of *S*. Enteritidis outbreak strains associated with shell eggs (Dallman et al., 2016; Inns et al., 2017; Pijnacker et al., 2019), including near real-time settings (Inns et al., 2015). More recently, the trend toward the adoption of culture-independent foodborne pathogen detection, the use of metagenomics in foodborne outbreak settings has also garnered considerable interest (Forbes et al., 2017).

During September 2019, a cluster of suspected foodborne illnesses involving primarily young children have been reported at hospitals throughout two adjacent cities, Shenzhen and Dongguan, within the Guangdong province of China. We describe a rapid cross-jurisdiction foodborne outbreak response between the Shenzhen, Dongguan and Guangdong Centers for Disease Control and Prevention, coordinated with an unprecedented efficiency and scale in China, and complemented by a multicenter effort to conduct near real-time whole-genome and metagenomics analyses to establish the possible links between *S*. Enteritidis strains isolated from case-patients, food handlers, kitchenware, and the egg sandwiches that were implicated as the source of outbreak. Genomic source tracing was conducted to elucidate the transmission dynamics of the outbreak-associated *S*. Enteritidis clone in China.

## Materials and Methods

### Multidisciplinary and Integrated Public Health Emergency Response

**Figure 1:**
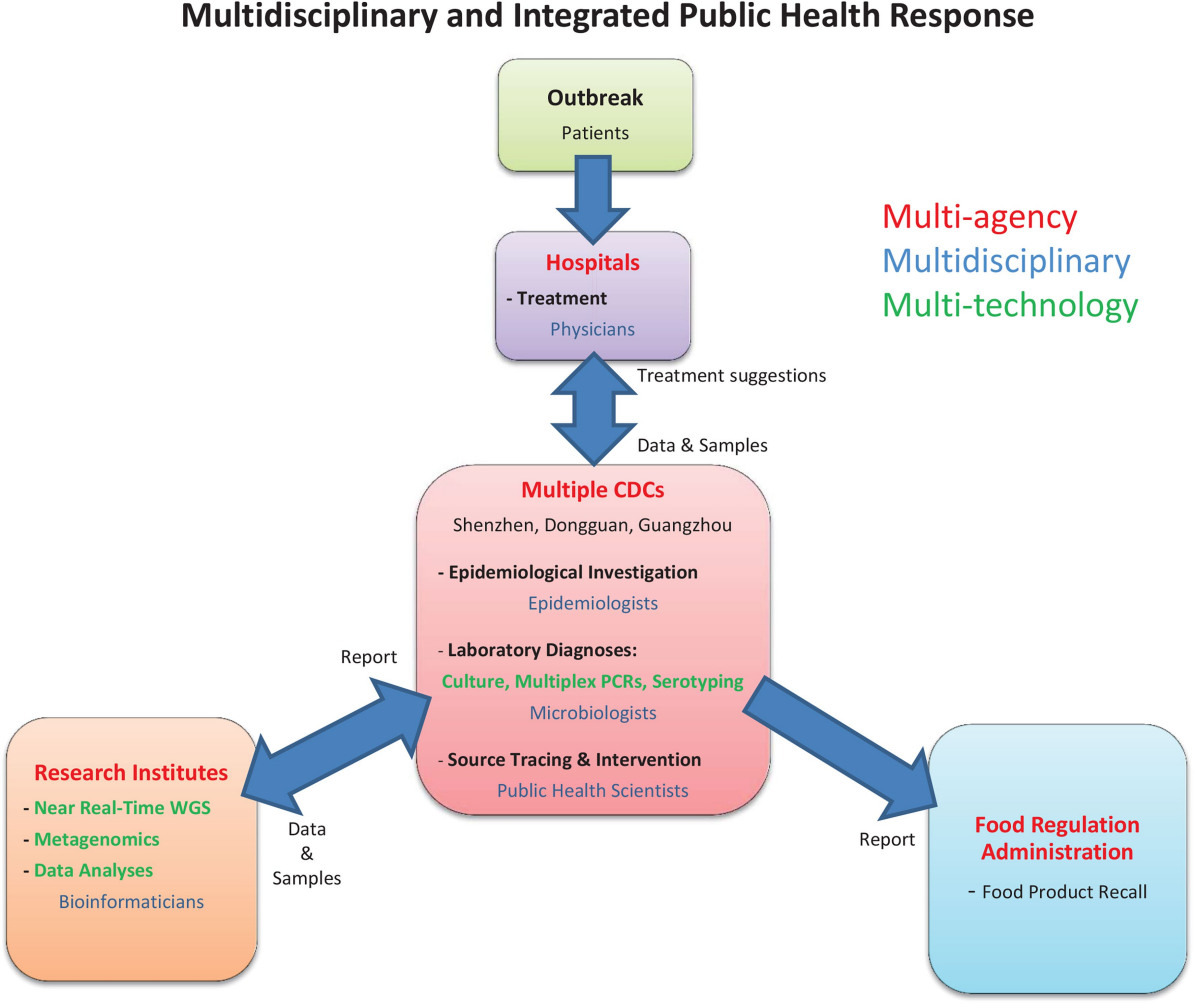
An overview of the multidisciplinary and integrated public health response framework. In the current *Salmonella* Enteritidis outbreak, the three CDCs (Shenzhen, Dongguan, and Guangdong) played a central role in coordinating a multicenter effort to conduct epidemiological investigation, laboratory diagnoses, near real-time whole-genome and metagenomics analyses, and genomic source tracing to stipulate intervention strategies.

A rapid cross-jurisdiction foodborne outbreak response was jointly coordinated by the Shenzhen, Dongguan and Guangdong Centers for Disease Control and Prevention, through a multicenter effort to conduct epidemiological investigation, laboratory diagnoses, near real-time whole-genome and metagenomics analyses, genomic source tracing, and stipulate intervention strategies, as summarized in Figure 1. Epidemiological investigations and analyses began on September 22 and were conducted by the Dongguan and Shenzhen CDCs. Dongguan CDC was also responsible for the on-site field investigation at the nursery, as well as the collection of samples from food (*n* = 19), the environment (*n* = 7), food handlers, and kitchen staff (*n* = 11). Samples from case-patients were collected by hospital physicians in Shenzhen (*n* = 46) and Dongguan (*n* = 30). Rapid diagnostic testing using a FilmArray GI panel multiplex PCR was performed at Shenzhen CDC. Bacterial culture, isolation, and identification were performed by Dongguan and Shenzhen CDCs during September 22–26. On September 26 and the ensuing 34 h, bacterial isolates were sent to Beijing Institute of Microbiology and Epidemiology (*n* = 14) and the Guangdong CDC (*n* = 52) for whole-genome sequencing, whereas metagenomic samples were sequenced by Shenzhen CDC (*n* = 30). Following the identification of the implicated food source, a traceback investigation was conducted by Shenzhen CDC and Shenzhen Agricultural Product Quality and Safety Inspection and Testing Center. All results generated were collated at Shenzhen CDC for data and bioinformatics analyses.

### Epidemiological Investigation

On-site investigation included interviews with school and kitchen staff, inspections of kitchen and dining areas of the canteen, reviewing surveillance video recordings, procedures of food preparation, and meal serving records. Demographic data, onset of illness, clinical symptoms, and food consumption histories were obtained through structured questionnaires collected at hospitals. During and immediately following the investigation period, the nursery was suspended from operation; pending detailed review of food preparation procedures at the nursery canteen. Standard epidemiological investigation procedures were followed, for which a summary workflow is provided as Supplemental Material (Supplementary Figure S1). A case was defined as a person with acute onset of three or more episodes of diarrhea and/or vomiting in a 24-h period, accompanied by fever >37°C or abdominal pain, who has consumed foods prepared by the nursery canteen since September 18. Active case finding was conducted by raising public health alerts to healthcare providers in an effort to identify additional persons throughout both cities whom met the case definition.

### Pathogen Identification

Samples collected (*n* = 113) included rectal swabs or stool samples from case-patients (*n* = 76) and food handlers (*n* = 11), food samples (*n* = 19), and kitchenware (cutting boards, serving plates, refrigerator, and mayonnaise mixing bowl; *n* = 7) from the nursery canteen. Stool samples from 10 cases with severe symptoms were processed using the FilmArray GI panel (BioFire Diagnostics, Salt Lake City) multiplex PCR as previously described (Hu et al., 2020). All samples were enriched and screened for common foodborne pathogens including *Salmonella*, *Shigella*, *Vibrio parahaemolyticus*, *Vibrio cholerae*, *Staphylococcus aureus*, *Escherichia coli* O157:H7, enterotoxigenic *Escherichia coli*, enteropathogenic *Escherichia coli*, enteroinvasive *Escherichia coli*, enterohemorrhagic *Escherichia coli*, *Bacillus cereus*, group A *Streptococcus*, and *Listeria monocytogenes*, as previously described (Hu et al., 2008). Food samples were sliced into small pieces aseptically, and 25 g was diluted in 225 ml enrichment broth and homogenized. Samples were enriched in selenite cystine broth (SC) at 35°C for 18–24 h, and a loopful then streaked into Hektoen Enteric (HE) agar plates, which were incubated at 35°C for 18–24 h. A minimum of five suspected colonies were picked and subjected to biochemical (Vitek 2 and bioMérieux) and serological tests for identification.

### Whole-Genome Sequencing and Metagenomic Analysis

DNA extraction was performed using the Qiagen QIAamp DNA Mini Kit (QIAGEN, Hilden, Germany) according to manufacturer’s instructions. Whole-genome and metagenomics sequencing were conducted in near real-time at sequencing facilities from multiple cities in China. WGS of outbreak isolates were performed at Beijing Institute of Microbiology and Epidemiology using Ion S5 or at Guangdong CDC using Illumina MiSeq, while historic strains from the Guangdong province were sequenced using Illumina MiSeq or BGISEQ-500. Metagenomics sequencing was conducted by BGI in Shenzhen using MGISEQ-2000. All sequencing data with uniform output formats were quality controlled and analyzed using same bioinformatics pipelines at Shenzhen CDC. Briefly, over 1GB of clean data were generated for each whole-genome sequenced isolate (>100x coverage, WGS) or metagenomics sample on average. Short-read sequence data were deposited in the NCBI Sequence Read Archive (BioProject PRJNA565566, accession numbers provided in Supplementary Table S1). Core-genome single-nucleotide polymorphisms (core-SNPs) were identified by the Snippy pipeline v4.3.8^1^ using *S*. Enteritidis reference genome P125109 (accession number: NC_011294) as previously described (Jiang et al., 2020). SNPs located in repetitive and recombinogenic regions were removed prior to phylogenetic analysis. Repetitive regions in the reference genome were identified using TRF v4^2^ and self-alignment by BLASTn, and recombinogenic regions were identified using Gubbins v2.3.4 (Croucher et al., 2015). The maximum likelihood trees were constructed based on non-repetitive and non-recombinogenic core-SNPs using IQ-TREE (Nguyen et al., 2015). Genomic source tracing was conducted by interrogating *S*. Enteritidis genomes (*n* = 241,991) from the Enterobase (Assessed September 2019; Zhou et al., 2020)^3^ for closely related strains (<10 core-genome multilocus sequence typing alleles differences) for inclusion into phylogenetic analysis. Pair-end libraries with short DNA fragments were constructed for whole-genome shotgun metagenomic sequencing by BGI in Shenzhen using MGISEQ-2000. After quality control, metagenomic sequencing reads were analyzed using Kraken v2.0.8 (Wood et al., 2019) for taxonomic classification and abundance estimates of *S. enterica* present in samples.

## Results

### Epidemiological Investigation

**Figure 2:**
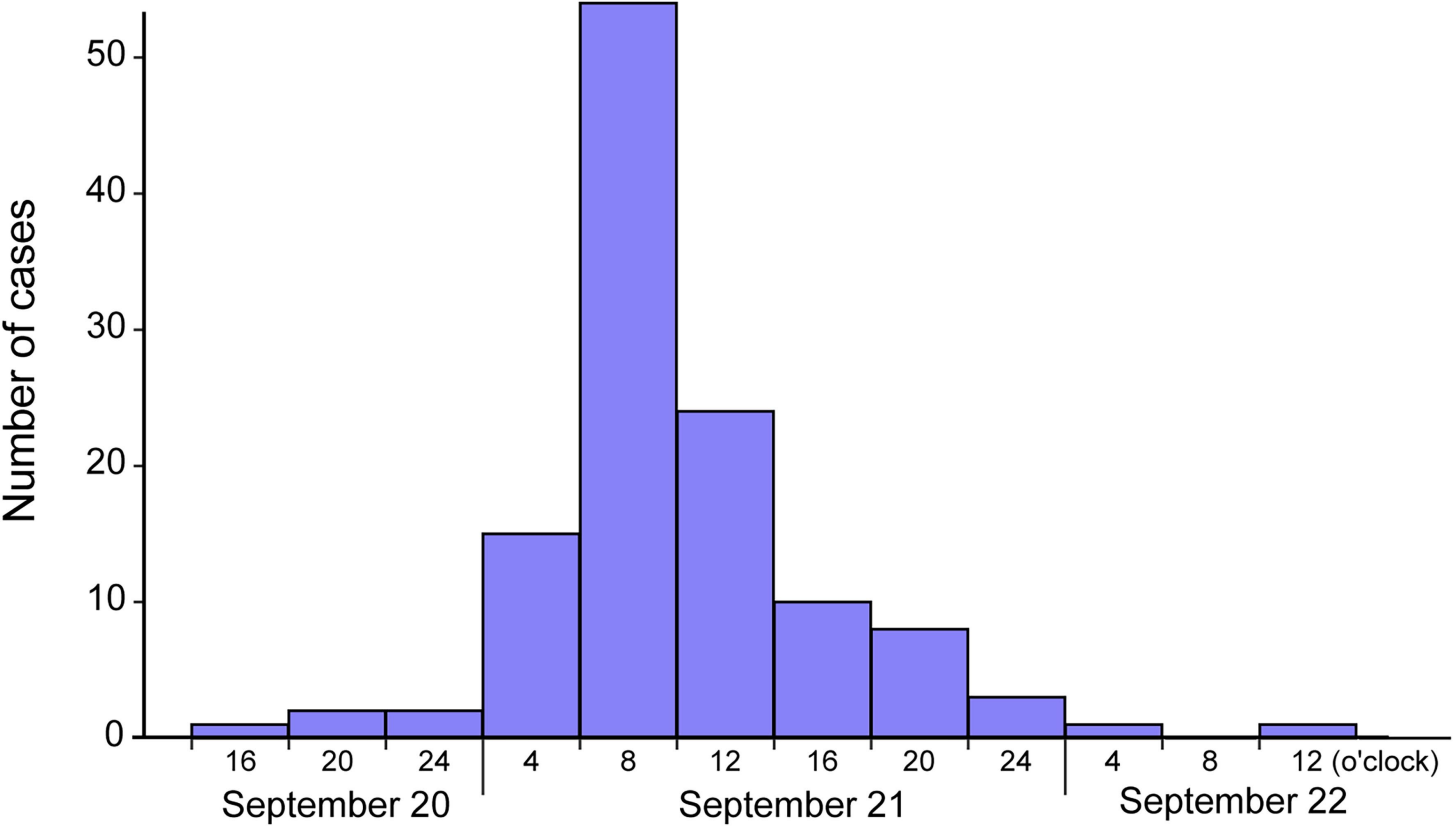
Epidemic curve of the *Salmonella* Enteritidis outbreak associated with the consumption of egg sandwiches in a nursery school, Shenzhen and Dongguan, Guangdong province, 2019.

Between September 20 and 22, a total of 254 cases of gastroenteritis were reported in Shenzhen and Dongguan, Guangdong province, China. Preliminary investigation indicated that only those who were either children or staff from a local nursery school in Dongguan located near the city border with Shenzhen had become sick. Of 157 persons who met the outbreak case definition during initial epidemiological investigation and active case finding, demographic data and exposure questionnaires were obtained for 121 case-patients and were included in this study. The age range of cases was 2–61 years, which included young children (age 2–6; *n* = 116), teaching staff (*n* = 3), and relatives of teaching staff who consumed leftovers brought home (*n* = 2), with a male to female ratio of 61–39%. Predominant clinical manifestations included fever (98.4%), diarrhea (89.3%), abdominal pain (87.6%), and vomiting (79.3%). One case presented with severe diarrhea, vomiting, and a fever of 39°C was admitted to intensive care unit, none died. The distribution of cases over 3 days (September 20–22) from the epidemic curve suggested a point-source outbreak with a single incubation period, which peaked on September 21 between 4 am and 8 am with 54 cases (Figure 2).

On-site investigation conducted on September 22 found that the disease prevalence among children attending full-day classes and half-day classes at the nursery were 25.84% (116/449) were 0 (0/18), respectively, which was significantly different (*χ*^2^ = 4.88, *p* < 0.05). Children from half-day classes were picked up at noon and subsequently were not given lunch and afternoon snacks. Combined with the time distribution of the cases, it is considered that the lunch or afternoon snack in the nursery canteen on September 20 may have led to the outbreak. Different foods were provided for teaching staff and children at the nursery, and no sandwiches were served for teaching staff on September 20. However, three sick teaching staff and two of their relatives who consumed leftovers sandwiches brought home developed symptoms of gastroenteritis and were eventually identified as cases. Interviews conducted found that mayonnaise containing raw eggs was spread directly on to bread, made into sandwiches, and served without heating. Based on these epidemiological findings, egg sandwiches served to children on September 20 as afternoon snacks were implicated as the suspected food source of the outbreak.

### Pathogen Identification

From a total of 113 samples, 66 were positive for *S*. Enteritidis, which were isolated from case-patients (*n* = 58), food samples (*n* = 4), food handlers (*n* = 2), and the mayonnaise mixing bowl (*n* = 2). Of the 10 samples tested by the FilmArray GI panel multiplex PCR, nine were found to be positive for *Salmonella*, while only seven samples had suspected colonies on HE plates. Using the Vitek 2 rapid diagnostic system, suspected colonies were identified to be *Salmonella* species, while serological testing has been determined as serotype Enteritidis.

### Whole-Genome and Metagenomic Analysis

**Figure 3:**
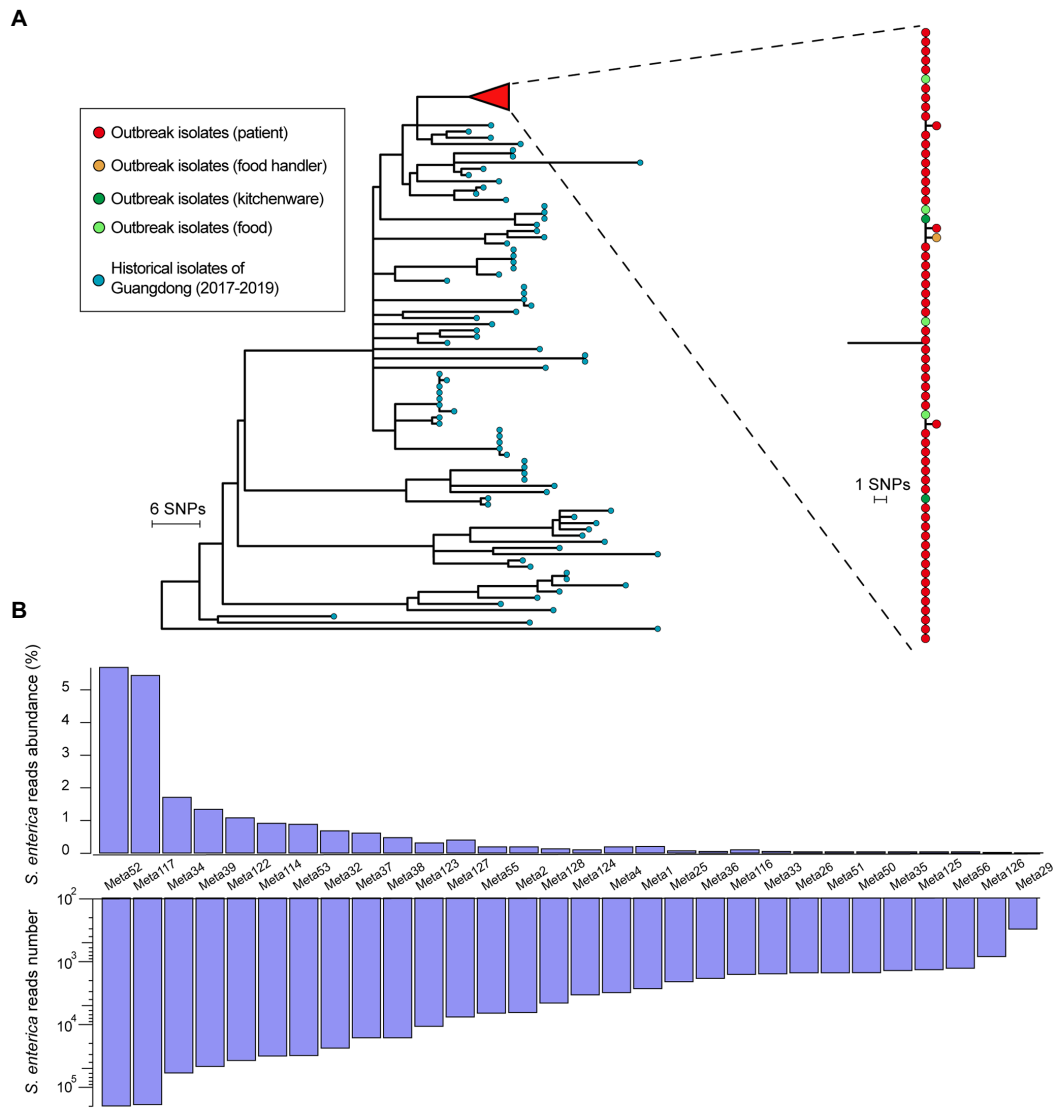
Whole-genome and metagenomic analysis of *Salmonella* Enteritidis outbreak isolates and historical isolates. **(A)** Maximum likelihood tree of outbreak isolates (*n* = 66) and historical isolates from Guangdong (*n* = 81) between 2017 and 2019. Colored circles in the tree tips indicate isolate source. **(B)** Distributions of species-specific abundances (top) and number of reads assigned to *Salmonella enterica* among metagenomics samples (*n* = 30).

The phylogenetic relationships inferred by WGS SNP-based cluster analysis between all sampled isolates were concordant with epidemiological findings. A maximum likelihood tree was constructed to visualize that all outbreak-associated isolates were genetically closely related to each other (Figure 3A). From a total of 860 core-SNPs identified, all outbreak-associated *S*. Enteritidis isolates differed by pairwise distances of one SNP or less and clustered together, including isolates from sandwiches, the mayonnaise mixer, and case-patients (SNP distance matrix provided in Supplementary Table S2). In conjunction with epidemiological analyses, the kitchen-made mayonnaise used as an ingredient of egg sandwiches was determined to be the source of the outbreak. In comparison, the minimum distance between historic isolates from the Guangdong province (*n* = 81) and any of the outbreak-associated isolates was 14 SNPs, larger than the common threshold (≤3 SNPs) used for delineating outbreak clusters (Taylor et al., 2015), suggesting a non-local outbreak source. From the preliminary metagenomics analysis of patient fecal samples conducted, 300–197,790 reads (median: 3,967) were assigned to *S. enterica*, with species-specific abundances for *S. enterica* ranked within top 10 of all abundances for 50% of samples (*n* = 15), ranging from 0.01 to 5.69% (median 0.19%) per sample (Figure 3B).

### Genomic and Epidemiological Source Tracing

**Figure 4:**
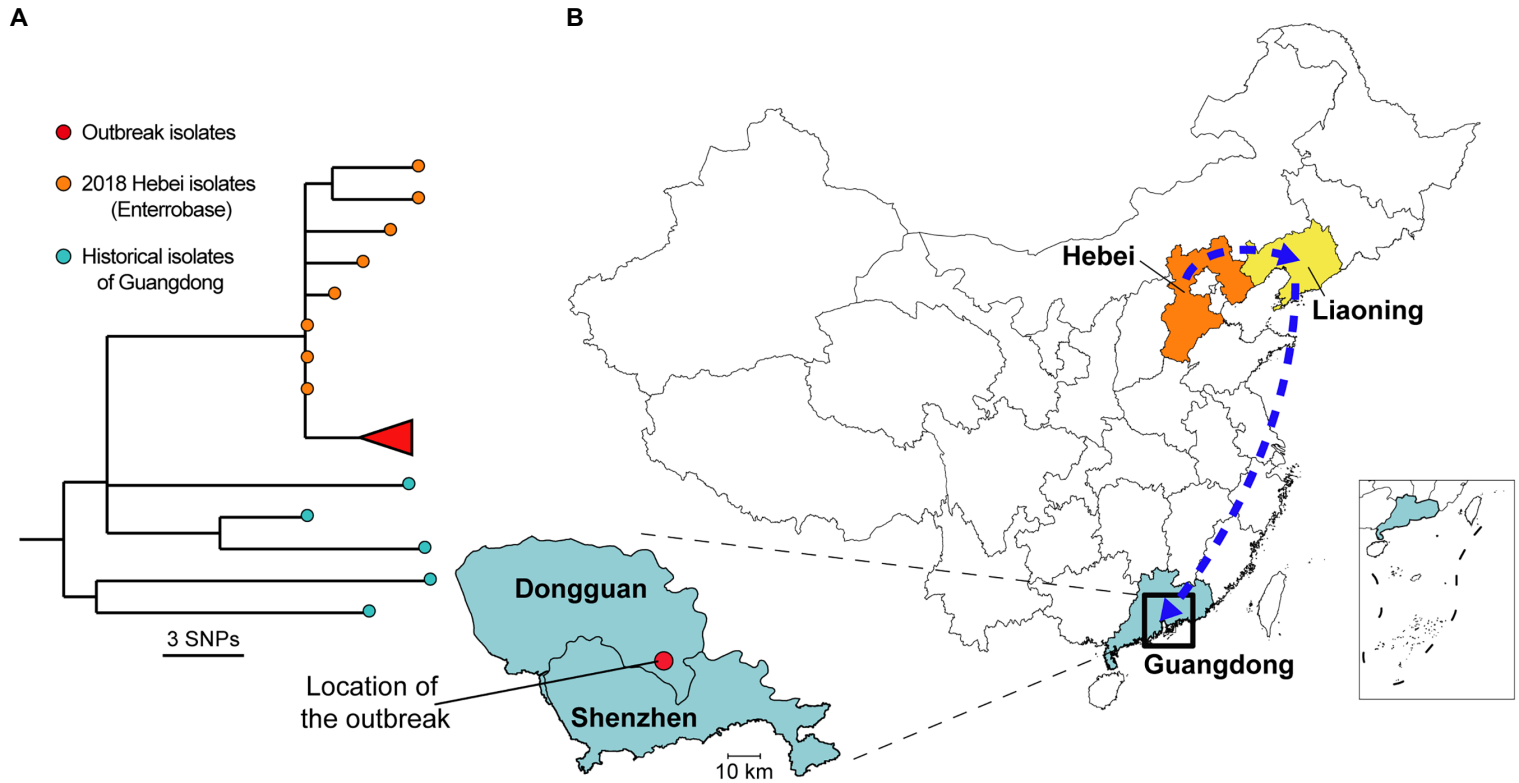
Possible transmission dynamics of the *Salmonella* Enteritidis clone throughout a multi-provincial egg distribution network in China. **(A)** Maximum likelihood tree of outbreak isolates (*n* = 66), historical isolates (*n* = 5, SNP distance < 22), and Enterobase isolates (*n* = 8, cgMLST loci difference < 10). Colored circles in the tree tips indicate geographical origins corresponding to the map in the right panel. **(B)** Inferred transmission dynamics for the possible origins of a *S*. Enteritidis clone implicated in the current outbreak. This clone appeared to have originated from an egg producer in the Hebei province, with a possible transmission chain that included intermediary distributors in the Liaoning province, before being sold at wholesale markets in Dongguan, Guangdong province, which in turn gave rise to the current disease outbreak in Dongguan.

To further elucidate the possible origins of a non-local outbreak source, a genomic source tracing analysis conducted using the Enterobase (Zhou et al., 2020). A *S*. Enteritidis clone that shared the same SNP genotype (pairwise distance ≤ 2 SNP between genomes) with isolates from the current outbreak (Figure 4A) was found throughout a multi-provincial egg distribution network in China (*n* = 8), thereby revealing the possible transmission dynamics (Figure 4B). This clone appeared to have originated from an egg producer in the Hebei province, with a possible transmission chain that included intermediary distributors in the Liaoning province, before being sold at wholesale markets in Dongguan, Guangdong province, which in turn gave rise to the current disease outbreak in Dongguan. With the assistance from the State Administration for Market Regulation (SAMR), epidemiological traceback investigation revealed and confirmed that eggs used for mayonnaise production in this outbreak were purchased from the Dalingshan market, Dongguan, which was sourced from an egg distributor in Anshan, Liaoning Province, for a Hebei chicken farm.

## Discussion

This is the first report of a *S*. Enteritidis outbreak that affected a large number of children in China, which was met with an unprecedented outbreak response in the country, both in scale and speed, involving the use of multidisciplinary and integrated approaches.

A noteworthy aspect of the current outbreak was the swift identification of the outbreak source to be the kitchen-made mayonnaise used within egg sandwiches on September 20. This was achieved by conducting quick laboratory diagnoses, concurrently with exhaustive epidemiological, laboratory, and genomic investigations (Figure 1). First, the large number of sick individuals who were young children under the constant care of family members had promptly sought medical care and underwent rapid laboratory diagnosis based on the use of multiplex PCR. This has helped guiding the epidemiological investigation by limiting its scope in the early stages of the outbreak. Second, the detailed review of food preparation protocol and record of meals served has allowed the accurate pinpointing of mixing bowl that was used during mayonnaise preparation. Third, through food and environmental sample, collection, including food samples kept from before the outbreak had occurred, has allowed prompt bacterial culture and isolation to confirm the vehicle of disease transmission and whole-genome analysis. Finally, by applying whole-genome SNP-based cluster analysis in near real-time, we were able to rapidly establish the confirmatory link between *S*. Enteritidis isolated from case-patients, food handlers, kitchenware, and the mayonnaise used in sandwiches to provide the crucial confirmation for the outbreak source. However, the relatively short duration of outbreak has not allowed time for genetic mutations to occur among isolates, which could have been useful for determining the direction of transmission between food handlers and food source. Nonetheless, this highlighted the advantages of complementing traditional epidemiological investigations with WGS analysis to present definitive genomic evidence linking suspected food sources to infections. In this investigation, an unprecedented multicenter genome sequencing effort was deployed in three different cities in China (Shenzhen, Guangzhou, and Beijing) using three different sequencing platforms to complete whole-genome sequencing for all isolates within 34 h.

Based on the further observation that the minimum pairwise distance between the genomes of historic isolates and outbreak-associated isolates was at least 14 SNPs, which indicated a non-local outbreak source, we performed a genomic source tracing analysis by comparing existing genome sequences from one of the largest the public domain for *Salmonella* genomes, the Enterobase (Zhou et al., 2020). The subsequent finding of a match with a *S*. Enteritidis clone throughout a multi-provincial egg distribution network in China was unexpected. However, without cooperation from the egg producer, intermediary distributors, and wholesalers, we were unable to obtain actual samples or isolates. In addition, we proactively pursued the relevant CDCs from these regions for further information and isolates, but none could be obtained. Some prospective outbreaks from these geographical areas have been identified and examined, but none could be matched. Therefore, we could not further establish the definitive transmission pathway in the supply chain. The lack of data sharing and communication channels is a common problem of foodborne disease investigations and surveillance in China. Currently, no enforcement agency in China has vested jurisdictional authority for compliance from food producers and sellers during foodborne disease outbreaks, which has hindered efforts to prevent subsequent outbreaks (Tang et al., 2015). Nonetheless, we have illustrated from the current study as an example that it is indeed possible to collaborate in a foodborne outbreak investigation to achieve a successful outcome.

The potential utility of metagenomics analysis in a large outbreak situation to guide investigation efforts was also explored. Given that *S. enterica* was detected in all stool samples tested, the metagenomics approach could potentially serve as an effective rapid screening tool when combined with epidemiological evidence to assist in time-sensitive outbreak situations. One powerful advantage of the metagenomics approach is the use of direct sequencing from patient specimens (Forbes et al., 2017), which would significantly shorten the turnaround time by eliminating the need for days of bacterial culture to obtain pure isolates, as in the case for WGS. Indeed, several studies have examined the use of metagenomics for investigating severe foodborne outbreaks of *Salmonella* serotype Heidelberg (Huang et al., 2017) and Shiga-Toxigenic *E. coli* (STEC) O104:H4 (Loman et al., 2013), and explored the potential for additional applications such as the rapid identification of virulence and resistance genes, respectively. These studies, along with our findings, suggest a promising potential of metagenomics to accurately identify the causative agents from outbreaks. Challenges remain, however, including limitations related to sensitivity issues, computational intensiveness, and high costs (Allard et al., 2018; Besser, 2018) that have precluded its routine use thus far (Armstrong et al., 2019).

*Salmonella* Enteritidis outbreaks have continued to occur worldwide despite frequent report and many possible intervention strategies have been suggested. The current outbreak has highlighted the importance of basic kitchen hygiene in disease prevention, and the food safety challenges posed by practice of using raw egg-based ingredients in food preparation, especially in a nursery setting. This can be achieved by strengthening the food safety training and supervision for food service providers and/or caterers at nurseries, such as the use of pasteurized egg products or avoid recipes using raw eggs, which should be fully cooked. Hygiene measures included hand washing and the use of gloves before handling food, whereas raw and cooked foods are processed and stored separately to avoid cross-contamination. The implementation of effective and basic food safety education as a public health priority would reduce the opportunities of recurring *S*. Enteritidis outbreaks.

In this cross-city *Salmonella* outbreak, we illustrated the importance of efficient public health intervention that was facilitated through close collaboration and coordination between primary care physicians, epidemiologists, microbiologists, and bioinformaticians across jurisdictions and agencies, which successfully limited the scale and propagation of a large disease outbreak. By using multidisciplinary and integrated approaches in an effective and efficient manner that was unprecedented in China, our experience could potentially serve as a pragmatic model for public health responses to large-scale outbreaks of infectious diseases in the future.

## Data Availability Statement

The datasets presented in this study can be found in online repositories. The names of the repository/repositories and accession number(s) can be found in the article/**Supplementary Material**.

## Ethics Statement

Ethical review and approval were not required because sample and data collection of patients were done as part of the routine infectious disease surveillance.

## Author Contributions

MJ, QZ, YC, and QH conceived and designed the study. MJ, CY, PK, LZ, HF, YJ, LS, HC, BL, QC, YW, YG, YS, ML, XS, JL, LJ, RC, YD, and QS performed the experiments and data analysis. MJ, CY, and PK wrote the original draft. MJ, CY, YC, RY, and QH reviewed and revised the paper. All authors contributed to the article and approved the submitted version.

## Funding

This research was supported by the National Key Research & Development Program of China (no. 2017YFC1603902), Sanming Project of Medicine in Shenzhen (no. SZSM201811071), the China National Science and Technology Major Projects Foundation (no. 2017ZX10303406 and 2018ZX10714002), the National Natural Science Foundation of China (no. 81673174), and Shenzhen Key Medical Discipline Construction Fund (no. SZXK064).

## Conflict of Interest

The authors declare that the research was conducted in the absence of any commercial or financial relationships that could be construed as a potential conflict of interest.

## Publisher’s Note

All claims expressed in this article are solely those of the authors and do not necessarily represent those of their affiliated organizations, or those of the publisher, the editors and the reviewers. Any product that may be evaluated in this article, or claim that may be made by its manufacturer, is not guaranteed or endorsed by the publisher.

## Footnotes

^1^ https://github.com/tseemann/snippy

^2^ https://tandem.bu.edu/trf/trf.html

^3^ https://enterobase.warwick.ac.uk

## Supplementary Material

The Supplementary Material for this article can be found online at: https://www.frontiersin.org/articles/10.3389/fmicb. 2022.772489/full#supplementary-material

Supplementary Figure S1 The work flow chart for the epidemiological investigation of foodborne disease outbreaks in China.
