# Supplementary figures and images for: Rapid Multilateral and Integrated Public Health Response to a Cross-City Outbreak of Salmonella Enteritidis Infections Combining Analytical, Molecular, and Genomic Epidemiological Analysis
Source: Front Microbiol. 2022 May 4;13:772489. doi: 10.3389/fmicb.2022.772489 (PMC9117964; doi:10.3389/fmicb.2022.772489)

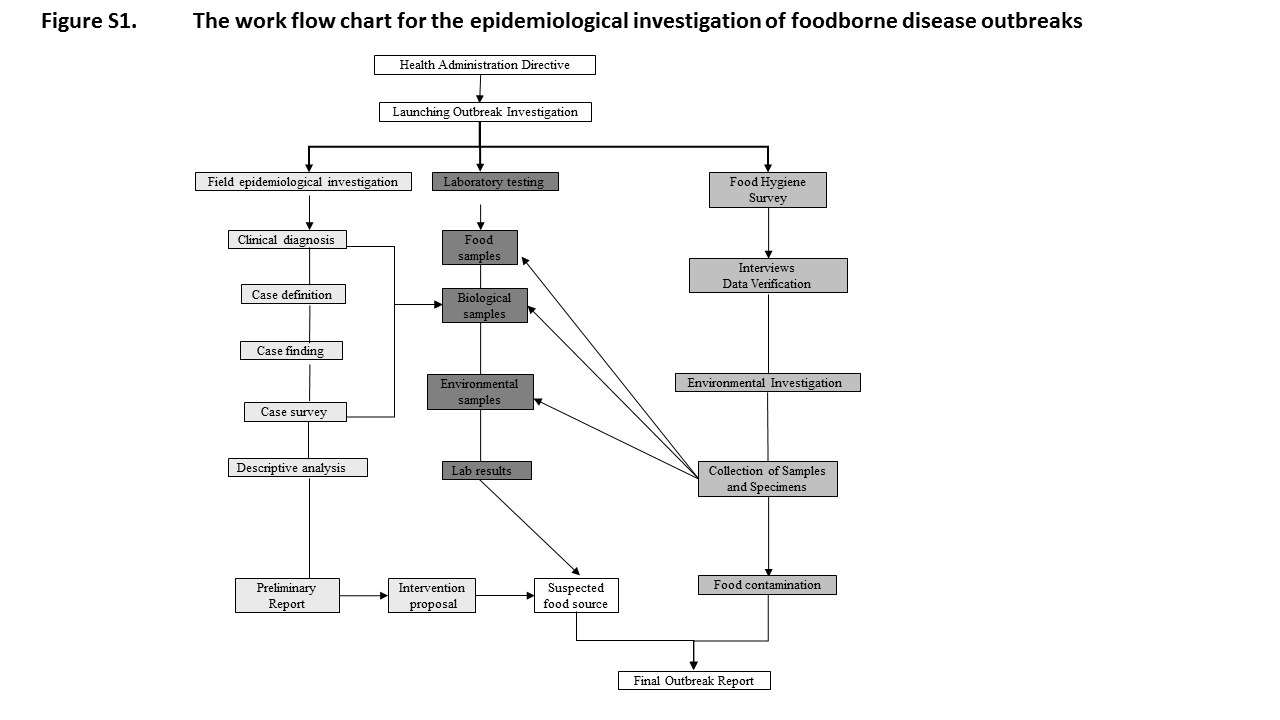

Supplement: Supplementary Figure S1 — The work flow chart for the epidemiological investigation of foodborne disease outbreaks in China. [file Image_1.tif]
